# Uncovering Work-Related Issues Among Canadian Organ Donation Coordinators: A National Mixed-Methods Study

**DOI:** 10.3390/healthcare14213738

**Published:** 2026-11-03

**Authors:** Vanessa Silva e Silva, Ken Lotherington, Michelle Lamont, Sonny Dhanani, Ewa Sucha, Richard Webster, Kendra-Lee Dupuis, Amina Silva

**Affiliations:** 1 School of Nursing, Brock University, St. Catharines, ON L2S 3A1, Canada; kdupuis@brocku.ca (K.-L.D.); asilva@brocku.ca (A.S.); 2 Canadian Blood Services, Ottawa, ON K1G 4J5, Canada; ken.lotherington@blood.ca (K.L.); michelle.lamont@blood.ca (M.L.); 3 Children’s Hospital, Eastern Ontario Research Institute, Ottawa, ON K1H 8L1, Canada; sdhanani@cheo.on.ca (S.D.); esucha@cheo.on.ca (E.S.); rwebster@cheo.on.ca (R.W.)

**Keywords:** organ donation, organ donor coordinators, occupational stress, burnout psychological, compassion fatigue, workforce

## Abstract

**Highlights:**

**What are the main findings?**

- This national mixed-methods study provides a comprehensive examination of work-related issues among organ donation coordinators in Canada, combining survey data from 120 coordinators with qualitative interviews involving 39 participants.
- Canadian organ donation coordinators reported moderate levels of burnout and compassion fatigue, alongside moral distress, with emotional exhaustion emerging as a factor associated with having considered leaving the role; 70% of participants had considered leaving their role at some point in their career.

**What are the implications of the main findings?**

- Organizational and policy-level strategies, including improved workload distribution, adequate staffing, and standardized program structures, warrant consideration and prospective evaluation as potential approaches to support coordinators’ well-being.
- Training that promotes self-awareness, coping, resilience, and social support may help coordinators recognize and manage work-related strain while strengthening the sustainability of organ donation programs.

**Abstract:**

**Objectives:** To investigate work-related issues and associated factors among organ donation coordinators in Canada. **Methods:** An explanatory sequential mixed-methods (QUAN-qual) design was used, incorporating a cross-sectional survey investigating demographic data, burnout in the work–life context, professional quality of life, and moral distress, and semi-structured qualitative interviews. **Results:** Of 175 eligible coordinators in Canada, 120 responded to the survey, and 39 were interviewed. The sample was predominantly female (n = 98; 82.4%) and composed mostly of registered nurses (n = 117; 97.5%). The average age of respondents was 41.8 years (Standard Deviation = 9.64). Survey findings indicated high levels of emotional exhaustion, moderate levels of depersonalization, moderate levels of burnout, and moderate levels of compassion fatigue. Notably, 70% (n = 84) of participants reported having considered leaving their role. **Conclusions:** This study’s findings highlight that Canadian organ donation coordinators experience moderate levels of burnout and compassion fatigue, alongside moral distress and substantial consideration of leaving the role. The qualitative findings further identified organizational, workload-related, and ethical contexts in which these experiences occur. Together, these findings highlight occupational strain among Canadian organ donation coordinators and support further investigation of organizational and workforce approaches to coordinator well-being.

## 1. Introduction

Transplantation can significantly improve the quality of life of patients facing end-stage organ failure while also reducing long-term healthcare costs [1,2]. Since most organ transplants rely on deceased donors, which is a highly complex and specific field, the success of deceased donation depends on the effective management of processes ranging from donor screening to organ recovery and allocation. These processes are carefully orchestrated by organ and tissue donation coordinator nurses (OTDCs), hereafter referred to as coordinators [3,4]. Coordinators, who are often critical care nurses, play a crucial role in delivering care during the deceased organ donation process, facilitating communication, testing, preservation, and transplantation, among other key tasks [5,6,7,8].

The complex organ donation process is accompanied by time-sensitive requests, last-minute schedule changes, and highly emotional encounters with donor families. The constant exposure to stressors and emotionally charged situations can leave coordinators susceptible to work-related issues, such as burnout (BO), compassion fatigue (CF), and moral distress (MD). MD refers to the psychological distress experienced when professionals are prevented from taking the actions they consider ethically appropriate due to organizational, institutional, or situational barriers [9]. BO is associated with chronic workplace stress and is characterized by emotional exhaustion, whereas CF reflects the emotional strain associated with repeated exposure to others’ suffering [10,11]. These work-related issues can negatively impact turnover and retention, as well as psychological health [12,13,14,15]. Additionally, existing evidence indicates that work-related problems, such as burnout, can permeate the workplace and affect other professionals, negatively influencing peers’ work-related well-being and job performance [16].

Challenges faced by coordinators, combined with the incidence of work-related issues [17], may prevent Organ Donation Organizations (ODOs) from reaching their full potential in deceased donation. When it comes to research and interventions, some local attention has been given to the mental health support required for this role through the ODOs. Still, there is currently no formal, standardized, national mental health support or baseline training available in Canada that focuses on the specific needs of Canadian coordinators [18]. Previous Burnout and Resilience in Organ Donor Coordinators (BRiC) publications have examined specific aspects of OTDCs’ experiences, including resilience and compassion satisfaction, the impact of the COVID-19 pandemic, and experiences related to complex donation cases [19,20,21,22,23]. The present study extends this work by examining a broader range of work-related issues among Canadian OTDCs, including burnout, compassion fatigue, moral distress, and turnover intention, as well as factors associated with these outcomes.

## 2. Materials and Methods

### 2.1. Design

This study is part of the BRiC research program, which aims to investigate and address work-related issues among coordinators in Canada. The first phase involved a scoping review of the global literature, which revealed a lack of studies and interventions on work-related matters despite coordinators’ vulnerability to them [8]. We used an explanatory sequential mixed-methods (QUAN-qual) design, in which the quantitative phase was conducted first and given priority in addressing the study aims. Findings from the quantitative survey informed the development of the semi-structured interview guide, allowing the subsequent qualitative phase to further explore and contextualize the quantitative findings. Quantitative and qualitative findings were integrated during the interpretation phase [24]. In other words, we conducted an online survey and followed up with recorded interviews with participants who expressed interest in participating. We followed structured, formal reporting methods, such as the STROBE [25] (Strengthening the Reporting of Observational Studies in Epidemiology) for cross-sectional studies and the COREQ [26] (Consolidated Criteria for Reporting Qualitative Research). Lastly, this study received ethics clearance from the Research Ethics Board at the Children’s Hospital of Eastern Ontario (20210076; 28 April 2021), and additional approvals were obtained at specific sites as needed; participants were provided with an informed consent form, and all coordinators consented to participate.

### 2.2. Setting and Sample

In this national investigation, coordinators from all Canadian ODOs were invited to participate in our study, which included those in the provinces of Alberta, British Columbia, Manitoba, New Brunswick, Newfoundland and Labrador, Nova Scotia, Ontario, Quebec, and Saskatchewan. Participants were recruited using a purposive sampling approach [27], and all coordinators were invited to participate via email. Eligible participants included healthcare professionals serving as coordinators who worked with deceased multiorgan donation in any Canadian province. Based on data shared by ODO representatives, the total available sample size at the time of data collection was 175 coordinators across Canada. The recruitment and data collection strategies occurred between January 2022 and March 2023.

### 2.3. Quantitative Methods

#### 2.3.1. Data Collection

The quantitative portion was done using a cross-sectional survey design approach. This approach allowed data collection at a single point in time, enabling analysis of variables. We collected demographic information to characterize our sample (e.g., age, gender, education), and used validated instruments to evaluate BO, CF and MD. The validated instruments included the Maslach Burnout Toolkit™ (Maslach Burnout Inventory—Human Services Survey for Medical Personnel—MBI-HSS-MP, the Areas of Worklife Survey—AWS), the Professional Quality of Life Measure (ProQOL), the Measure of Moral Distress for Health Care Professionals (MMD-HP) [28], and the 10-item Connor-Davidson Resilience Scale (CD-RISC-10) [29], which are described in Supplementary Material Table S1. MBI-HSS-MP scores were interpreted using the traditional cut-off values described in the MBI Manual (3rd ed.) [30], while ProQOL-5 scores were interpreted according to the scoring thresholds provided in The Concise ProQOL Manual (2nd ed.) [31]. The specific cut-off values used to classify scores as low, moderate, or high are provided in Supplementary Material (Tables S5 and S6). It is important to note that while these study measures offer valuable insights into work-related issues for coordinators, they are not intended to diagnose any associated conditions. Data on resilience (CD-RISC-10) and compassion satisfaction (ProQOL-CS) from this study population have been previously reported in a publication focused specifically on resilience among Canadian OTDCs [20]. The present study reports these measures in the context of a broader analysis of work-related issues, alongside burnout, secondary traumatic stress, moral distress, and turnover intention, which were not the focus of the previous publication.

#### 2.3.2. Procedures

The survey was hosted and administered online by Canadian Blood Services, Canada’s national agency for Organ and Tissue Donation and Transplantation (OTDT), to improve system performance and interprovincial organ sharing, using a third-party licensed product (Interceptum, Ottawa, ON, Canada) approved by their information technology (IT) and legal departments. Participants received an e-mail invite from the managers of their respective Organ Donation Organizations, and Canadian Blood Services had no influence on the decision of Organ Donation Organizations to support the study deployment; they accessed the survey and consented to participate before seeing the questions. Participants had 60 days to complete the study, and reminders were sent every 15 days. To preserve the anonymity of survey responses, contact information required for gift card distribution and for participants who expressed interest in the qualitative phase was collected through a separate form that was not linked to survey responses, and interviewers did not have access to participants’ survey responses. All participants who completed that survey received a CAD $50 gift card.

#### 2.3.3. Data Analysis

Results were imported into R Software Version 4.4.2 for statistical analysis [32]. Descriptive statistics were used to characterize sample demographics and scale scores; continuous variables were summarized using means, standard deviations, medians, and ranges, while categorical variables were reported as frequencies and percentages. Missing data were documented for each variable and reported alongside descriptive summaries; no imputation was performed given the low proportion of missing values across most measures.

Bivariate analyses were conducted to examine differences in scale scores across demographic and occupational subgroups. Independent samples *t*-tests (Welch’s test, not assuming equal variances) were used for two-group comparisons. To control the false discovery rate arising from multiple comparisons, the Benjamini–Hochberg procedure was applied across the five outcome comparisons (emotional exhaustion, compassion satisfaction, resilience, secondary traumatic stress, and moral distress) within each grouping variable (sex and years working as an OTDC). Both unadjusted and adjusted *p*-values are reported, and inferences for these comparisons were based on the adjusted values. In addition, Cohen’s d statistic was used to quantify the magnitude of the effect.

Pearson correlation coefficients were computed to assess the direction and magnitude of associations between continuous scale scores, with effect sizes interpreted as small (r = 0.1–0.3), medium (r = 0.3–0.5), or large (r > 0.5).

Prior to regression modelling, intercorrelations among predictor variables were examined to assess multicollinearity (Supplementary Material, Table S2). Scales with a large correlation (r > 0.5, consistent with the benchmark above) were not entered simultaneously into the same model. The correlation between emotional exhaustion and secondary traumatic stress in the analytic sample was 0.49 (Supplementary Material, Table S2), just below this threshold; both were retained because they capture related but conceptually distinct aspects of work-related strain, and collinearity in the final model was additionally assessed using variance inflation factors. Binary logistic regression was used to identify factors associated with having ever seriously considered leaving the current OTDC position, with results expressed as odds ratios (OR), each adjusted for all other predictors in the model, and 95% confidence intervals (CI). The outcome variable, having ever seriously considered leaving the current position as an OTDC, was coded as 1 = Yes and 0 = No. Complete-case analysis was used, resulting in 112 participants included in the final model; eight participants were excluded due to missing data in at least one predictor. Given the limited number of participants in the “No” category of the outcome variable within the analytic sample (n = 35), the number of predictors in the final model was restricted to four (approximately nine participants in the smaller outcome category per predictor; 35/4 = 8.75) to reduce the risk of overfitting. The four predictors were selected based on their clinical relevance and their representation of key occupational and psychological domains examined in the study, including emotional exhaustion, resilience, secondary traumatic stress, and experience in the OTDC role. Model fit was evaluated using the log-likelihood, Akaike information criterion (AIC), Bayesian information criterion (BIC), Tjur R^2^, area under the receiver operating characteristic curve (AUC), and root mean square error (RMSE). Tjur R^2^ and AUC were used to assess model discrimination, while RMSE quantified prediction error. Lower AIC, BIC, and RMSE values and higher Tjur R^2^ and AUC values indicated improved model performance. Variance Inflation Factor (VIF < 2) was used to rule out multicollinearity between factors included in the model. VIFs ranged from 1.17 to 1.44. Statistical significance was set at *p* < 0.05 for all analyses. All quantitative analyses were performed by professional statisticians with experience in health research (R.W. and E.S.).

### 2.4. Qualitative Methods

#### 2.4.1. Data Collection

A descriptive approach was used for the qualitative portion of this study. Upon completing the quantitative survey, participants were asked whether they would be interested in participating in the qualitative component. Interviews with those who expressed interest were scheduled in the order in which they responded. This pragmatic approach was used because participation in the qualitative phase was voluntary and based on participants’ expressed willingness to be interviewed; no demographic quotas or stratified selection criteria were applied. The semi-structured interview guide was informed by findings from the quantitative survey, as well as by the study aims, key constructs investigated in the study, including BO in the work–life context, professional quality of life, and MD, and the existing literature on BO, CF, MD, and resilience among healthcare professionals. The guide was designed to further explore and contextualize findings from the quantitative phase through participants’ experiences. Open-ended questions were designed to encourage participants to describe their workplace experiences and the organizational and personal factors they perceived as contributing to these work-related issues. Follow-up probes were used, when appropriate, to encourage participants to elaborate on their responses. Prior to data collection, pilot interviews were conducted using the semi-structured interview guide (Supplementary Material, Table S3). No substantial revisions were required following the pilot interviews; therefore, these interviews were retained in the final analysis. Independent interviews were conducted online via Zoom^®^ video conferencing and recorded in both video and audio, lasting between 29 and 146 min. Interviews in English were conducted by a female member of the research team with experience in qualitative research (A.S., RN, PhD), and in French by a female research assistant with qualitative training (J.V.). The interviewer informed participants of their affiliation with the organization conducting the research and maintained a neutral approach throughout the interviews. Participation was voluntary, and participants were informed that their responses would remain confidential and would not be shared individually with their employer or ODO. The interviewers did not have a supervisory relationship with the participants. These measures were intended to minimize potential response and social desirability bias associated with the organizational affiliations of members of the research team. Within the qualitative phase, data collection and analysis occurred concurrently to enable ongoing assessment of data saturation. All 39 survey respondents who expressed interest in participating in the qualitative phase were interviewed. During data collection and concurrent analysis, recurring patterns were identified across participants’ accounts, and the later interviews were not generating substantively new information relevant to the study objectives. No predetermined numerical threshold for saturation was used; rather, saturation was assessed iteratively based on the recurrence of information and the adequacy of the data to capture the breadth and depth of participants’ experiences. Lastly, descriptive and reflexive notes taken during the interview process were included within our data analysis to give more context and enhance understanding.

#### 2.4.2. Data Analysis

Recorded interviews were transcribed verbatim by professional transcriptionists after completion, with all personal identifiers removed before analysis. Transcripts were not returned to participants for member checking. Interviews conducted in French were translated into English by a professional translator during transcription. All transcripts were imported into NVivo^®^ to organize and manage the data. Qualitative data were analyzed using a content analysis approach. One member of the research team (A.S.) read the transcripts repeatedly to gain familiarity with the data before conducting open coding. Codes with similar meanings were grouped into categories, which were iteratively reviewed and refined through constant comparison across interviews. Although the semi-structured interview guide was informed by findings from the quantitative phase, the study aims, and the key constructs under investigation, these constructs were not used as a predetermined coding framework. Thus, deductive elements primarily informed data collection, while the analytical process remained inductive and data-driven, with codes and categories developed from participants’ accounts. The coding framework and resulting categories were peer-reviewed by two additional members of the research team (A.R. and V.S.S.). Differences in coding or interpretation were discussed among the three researchers and resolved through consensus. Reflexive field notes and memos were maintained throughout data collection and analysis, while team-based discussions were used to support the credibility and dependability of the analytical process. Within the qualitative phase, interviewing, transcription, and data analysis occurred concurrently, allowing the research team to assess whether revisions to the interview guide were needed. In the Results section, all quoted pronouns have been replaced with gender-neutral pronouns (they/them) to protect participant confidentiality. Finally, quantitative and qualitative findings were integrated during the interpretation of the results to provide a more comprehensive understanding of coordinators’ work-related issues.

## 3. Results

### 3.1. Participant Characteristics

According to ODO reports collected at the time of data collection, the available population consisted of 175 coordinators across Canada. A total of 120 coordinators participated in the quantitative phase (response rate: 68.6%), and 39 in the qualitative phase. Most participants were registered nurses (RNs) (n = 117, 97.5%), with the remainder being respiratory therapists (RTs) (n = 3, 2.5%). Most had worked more than one year as coordinators (n = 99, 83.9%). The mean (SD) age was 41.8 (9.64) years, and most participants were female (n = 98, 82.4%). Among the 39 qualitative participants, the average experience as an OTDC was 6.9 years; 35 had a bachelor’s or master’s degree in nursing, 32 had a critical care background, and 20 worked full-time. Full demographic characteristics are presented in Table 1.

### 3.2. Burnout and Compassion Fatigue

BO was assessed using both the MBI-HSS-MP scale, which measures three domains of burnout (emotional exhaustion—EE, depersonalization—DP, and personal accomplishment—PA), and the ProQOL scale, which measures compassion satisfaction (CS), burnout (BO), and secondary traumatic stress (STS). Coordinators demonstrated, as measured by MBI-HSS-MP (Table 2), high levels of EE (mean = 28.4, SD = 11.2), moderate levels of DP (mean = 7.76, SD = 5.44), and moderate levels of PA (mean = 32.1, SD = 6.94). When evaluated using the ProQOL scale (Table 3), coordinators also reported moderate levels of BO (mean = 27.6, SD = 6.07).

Welch’s independent-samples *t*-tests were conducted to examine differences in the five study variables by sex (Table 4) and by years working as a coordinator (Table 5). We did not find any statistically significant difference in emotional exhaustion scores between males and females in our sample (Table 4), nor in years working as a coordinator (Table 5). However, female participants reported higher STS scores than male participants (27.3 [SD = 6.95] vs. 22.8 [SD = 7.63]), with a medium effect size (Cohen’s d = 0.639, 95% CI: 0.154–1.124). This difference was statistically significant before correction for multiple comparisons (unadjusted *p* = 0.019) but not after Benjamini–Hochberg adjustment (adjusted *p* = 0.094) (Table 4). Given this, and the small number of male participants (n = 21), this finding should be regarded as exploratory. Participants who had worked as an OTDC for more than one year had significantly higher CD-RISC-10 scores than those who had worked for one year or less (29.2 [SD = 5.47] vs. 24.9 [SD = 5.47]; unadjusted *p* = 0.005; Benjamini–Hochberg-adjusted *p* = 0.027), with a medium effect size (absolute Cohen’s d = 0.774, 95% CI: 0.255–1.292); that is, the average resilience score of coordinators with more than one year of experience was approximately 0.8 standard deviations higher than that of coordinators with one year or less of experience (Table 5). Pearson correlation analyses were conducted to examine the associations between age and those same variables. Correlations between age and study variables were small or negligible (|r| ≤ 0.15; Table 6). We also explored correlations between weekly hours working as coordinators and those same variables, and weekly hours as a coordinator showed a small positive association with emotional exhaustion, as indicated by a Pearson correlation coefficient of 0.25 (Table 7).

CF was analyzed using the ProQOL scale (Table 3), specifically the Secondary Traumatic Stress (STS) sub-domain. Coordinators experienced moderate levels of STS (mean = 26.4, SD = 7.24). In our *t*-test, years of experience were not statistically significant for STS experience in the study population (Table 5).

While many participants reported low self-identification with BO and CF, describing work-related strain as “part of the role” (P10) and an “expectation” (P10), most acknowledged exposure over time and described experiences that mapped onto BO/CF domains. Participants noted that BO and CF could develop “after… a few cases” (P1) or occur “in waves” (P33), and described emotional and physical impacts including “disconnect” (P28), “detachment” (P36), and “desensitization”(P25), as well as being tired “physically and psychologically” (P38), experiencing “fatigue” (P5), “insomnia” (P38), and “nightmares” (P17). Triggers described by participants clustered around work environment pressures (workload, long hours, staffing shortages) and role demands (multiple team expectations, competing priorities, emotionally difficult conversations).

### 3.3. Areas of Worklife

Discrepancies or gaps between workers’ expectations and their actual experiences in the workplace, along with the psychosocial conditions of work–life, were assessed using the Areas of Worklife Survey (AWS). Among the six areas of work–life measured using the AWS, workload (mean = 2.58, SD = 0.690) and fairness (mean = 2.92, SD = 0.808) were the only areas with mean scores below the AWS mismatch threshold of 3.0. Workload mismatch reflected perceptions of working beyond human limits, whereas fairness mismatch reflected perceived inconsistencies in organizational decision-making, resource allocation, and workplace procedures (Table 8). These mismatches were reflected in participants’ accounts, including being expected to work beyond their shift, expanded travel due to staffing, feeling overworked, and difficulty in finding break periods within their shift (Table 9).

### 3.4. Moral Distress

MD was assessed using the MMD-HP scale. Among participants with available MMD-HP data (n = 90), the mean MD score was 108 (SD = 80.8) (Table 10). Given the 25% missing data for this measure, this finding should be interpreted with caution. Participants who had worked as an OTDC for one year or less reported significantly higher MD scores than those who had worked for more than one year (173 [SD = 108] vs. 93.5 [SD = 66.5]; unadjusted *p* = 0.015; Benjamini–Hochberg-adjusted *p* = 0.037), with a large effect size (Cohen’s d = 1.043, 95% CI: 0.457–1.629); that is, the average MD score of coordinators with one year or less of experience was approximately one standard deviation higher than that of coordinators with more than one year of experience (Table 5). Participants described morally distressing and ethically challenging situations as uncomfortable, often characterized by a sense of being compelled to proceed with cases to comply with institutional policies. A commonly described care-related source of discomfort involved approaching families despite believing donation was unlikely to be successful based on their experience with the clinical status of the patient. Participants also reported discomfort with being expected to join end-of-life discussions and perceived fragmentation in the informed consent process. Organizational contributors included team pressure and resource constraints, such as staffing issues and operating room challenges, which were described as hindering consistent patient-centred care and contributing to late shifts and increased workload. In contrast, some participants denied experiencing MD, describing that making the right choices for the patient prevented such an occurrence. Table 11 contains representative quotes of the issues described above.

### 3.5. Job Turnover Intentions

Survey results indicated that a substantial proportion of coordinators had contemplated leaving their position at some point in their careers. As shown in Table 1, 70% (n = 84) of coordinators reported considering leaving their roles. In a multivariable logistic regression analysis including MBI-HSS-MP Emotional Exhaustion, CD-RISC score, ProQOL-STS score, and years working as an OTDC (n = 112), higher emotional exhaustion was significantly associated with increased odds of having considered leaving the OTDC position. Each one-point increase in the MBI-HSS-MP Emotional Exhaustion score was associated with a 14% increase in the odds of having considered leaving the OTDC position (OR = 1.14, 95% CI: 1.08–1.21; *p* < 0.001). Working more than one year as an OTDC was not significantly associated with having considered leaving: although the estimated odds were 3.24 times those of participants who had worked one year or less, the confidence interval was wide and included 1 (OR = 3.24, 95% CI: 0.88–12.7; *p* = 0.076) (Table 12).

This was echoed in participant reports, with turnover considerations described as occurring “every day” (P8), “frequently” (P30), or “a few times” (P1, P12). An overall feeling of “low morale” (P1) was emphasized, along with workload and the hours required. Some participants described applying for other jobs or telling a previous manager they “want to come back” (P4). Former coordinators who had left described it as an “easy decision” (P19) and that they “should have done it sooner” (P19), while also noting that some would consider returning if organizational factors such as staffing and scheduling improved. Others indicated they did not feel “stressed or strained” (P36) enough to leave (Table 13).

## 4. Discussion

This study provides a comprehensive national exploration of Canadian organ donation coordinators’ work-related challenges using validated measures and complementary qualitative inquiry. The findings indicate that coordinators experience moderate levels of BO and CF, alongside MD, with a high proportion of participants reporting that they had considered leaving their roles. This further emphasizes the seriousness of these issues and their potential impact on workforce stability and program sustainability. These findings are broadly consistent with a recent meta-analysis of nurses worldwide using the same ProQOL instrument, which reported similar levels of BO and STS. This suggests that, despite the unique ethical and logistical demands of organ donation coordination, the psychological burden experienced by coordinators is comparable to that reported across nursing populations [33].

Integration of the quantitative and qualitative findings suggests that the work-related burden identified in this study may be better understood as an interconnected occupational experience rather than as a set of isolated outcomes. Quantitative measures identified emotional exhaustion, secondary traumatic stress, moral distress, workload and fairness mismatch, and substantial turnover intention, while participants’ accounts provided context for understanding how these work-related issues were experienced in practice. In particular, participants’ accounts revealed recurring organizational and role-related pressures, including staffing constraints, extended working hours, competing responsibilities, and ethically challenging situations, which appeared across the qualitative findings related to burnout and compassion fatigue, work–life mismatch, moral distress, and decisions about remaining in the role. The qualitative findings therefore extend the quantitative results by suggesting that these outcomes may share overlapping organizational and role-related contexts, while also illustrating how coordinators interpret and navigate these pressures in their everyday work.

At the organizational level, scheduling frameworks and workload distribution represent potential targets for future interventions aimed at addressing emotional exhaustion. Training and interventions that foster coping, resilience, and social support may represent additional approaches warranting prospective evaluation in this workforce.

Results from this study highlight a complex array of work-related stressors reported alongside burnout, compassion fatigue, and moral distress among Canadian coordinators. Although effect sizes were small and causal inferences cannot be made, coordinators working more weekly hours tended to report higher levels of emotional exhaustion. While these findings suggest an association between workload and work-related strain, longitudinal studies are needed to determine whether increased working hours contribute to these outcomes. Taken together with the high turnover intentions reported in this study, these findings support consideration of scheduling frameworks and workload distribution as potential organizational targets for improving coordinator well-being. These considerations are directly informed by the workload and work-related stressors reported by participants. At a broader policy level, system-level investment, greater consistency in organ donation program structures, and adequate staffing may be considered as potential strategies to support the management of donation processes and coordinator well-being. These broader recommendations represent policy implications arising from the interpretation of our findings rather than direct conclusions of the study. Implementing such organizational changes may require broader system-level investment in organ donation programs.

The experience of BO, CF, and MD is prevalent among coordinators worldwide. Several factors associated with BO among coordinators in China align with those described by Canadian coordinators, including personal factors, job responsibilities, and donor-family factors [34]. Previous evidence suggests that training and education, as well as approaches supporting self-regulation, coping, and social engagement, may warrant further evaluation as potential strategies to support coordinators experiencing BO, CF, or MD [35,36].

One factor described by Canadian coordinators as being associated with these work-related issues in this study, and which should be explored further, was work-related conflict, which participants perceived as contributing to stress in their work environment. Transplant coordinators in Iran also reported being exposed to horizontal violence, described as stigmatization by colleagues expressed as insults and accusations of being unproductive [36]. Further exploration of conflict resolution strategies and workplace training may help address these experiences and support coordinators in performing their job responsibilities. Coordinators in this study also reported job responsibilities as perceived sources of stress. Supporting families, hospital teams, and transplant teams during the donation processes was described by participants as a source of stress and perceived burden. This suggests that further exploration of approaches to standardize organ allocation and communication processes may be warranted to support smoother processes and coordinator well-being.

The challenges associated with supporting donor families while coordinating the donation process may also reflect the inherently complex ethical nature of the coordinator role. Similar concerns have recently been discussed in the United States, where organ procurement organization professionals have been described as “dual advocates,” balancing responsibilities toward donor families while simultaneously supporting the interests of potential transplant recipients. This dual advocacy role places coordinators in ethically complex situations where competing obligations may not always be reconciled, creating conditions that contribute to moral distress and burnout [37]. Participants’ accounts further suggest that this burden may be compounded by organizational constraints, including staffing limitations and operating-room pressures, particularly when these constraints limit coordinators’ perceived ability to act in accordance with their professional or ethical judgment. These experiences align with the conceptualization of moral distress presented in the Introduction, whereby professionals may recognize an ethically appropriate course of action but perceive constraints on their ability to pursue it. Similarly, recent evidence among transplant providers identified a concerning prevalence of moral injury associated with the ethical complexity of transplantation, suggesting that exposure to morally challenging situations may persist despite formal training interventions [38]. Therefore, these findings reinforce that the psychological burden experienced by professionals involved in organ donation and transplantation may extend beyond workload alone and may also be associated with the ethical demands of the field.

Despite incidences of BO, CF, and MD among coordinators in this study, many reported not being able to self-identify these symptoms. Self-awareness has been reported in the literature as important for individuals to recognize stress and prevent burnout [39]. This finding is noteworthy, given the moderate to high levels of work-related issues identified in our study population. This discussion highlights the importance of knowledge of work-related issues for coordinators so they can recognize changes early and work to mitigate them, which should align with tailored training and support materials that emphasize not only aspects of organ donation processes, but also self-care and coping strategies.

An additional explanation may relate to the culture surrounding organ donation coordination. Participants frequently described emotional strain as “part of the role” or an “expectation,” suggesting that occupational stress may become normalized. While this culture may foster perseverance and commitment, it may also reduce recognition of early signs of burnout and compassion fatigue. This has been discussed in a separate publication part of the same research program as this paper, in which coordinators perceived themselves as resilient despite demonstrating lower resilience scores than other healthcare professionals [20]. Together, these findings suggest that resilience and dedication may coexist with an under-recognition of psychological distress, supporting further evaluation of approaches that promote both resilience and self-awareness.

## 5. Limitations

This study has limitations that should be considered when interpreting the findings. First, the cross-sectional design precludes establishing temporal or causal relationships between the variables examined. This is particularly relevant to the turnover analysis, as participants were asked whether they had ever seriously considered leaving their current OTDC position, whereas emotional exhaustion and other work-related measures reflected their current experiences. Therefore, the temporal relationship between current emotional exhaustion and prior consideration of leaving the role cannot be established. In addition, the logistic regression model included four predictors and 35 participants in the smaller outcome category among the 112 analyzed, corresponding to approximately nine participants in the smaller outcome category per predictor (35/4 = 8.75), which is slightly below the commonly cited guideline of ten; the model estimates, particularly the wide confidence interval for years of experience, should therefore be interpreted with caution.

Second, the quantitative findings were based on self-reported measures and may therefore be subject to response and social desirability bias. More broadly, voluntary participation in the study may have introduced selection or non-response bias. The sample was also predominantly composed of registered nurses and female participants, which may limit the extent to which the findings represent coordinators from other professional backgrounds or sex groups. In addition, MMD-HP data were missing for 30 participants (25% of the quantitative sample), resulting in an effective sample size of 90 for analyses involving moral distress. Complete-case analysis was used for these analyses. Participants with complete MMD-HP data (n = 90) were compared with those with missing MMD-HP data (n = 30) across available participant characteristics, with no substantial or statistically significant differences identified between the groups (Supplementary Material, Table S4). The reason for the missing MMD-HP data was not documented, and potential bias related to missing data cannot be entirely excluded.

Third, participation in the qualitative phase was voluntary among survey respondents who expressed interest in being interviewed, which may have introduced self-selection bias, as individuals with particularly strong experiences or perspectives may have been more likely to participate. Transcripts were not returned to participants for member checking, a deliberate decision by the research team to avoid adding to the workload of the study population, who were already under work strain. Interviews conducted in French were translated into English by a professional translator during transcription; therefore, some linguistic or contextual nuances may have been altered during translation. Although some members of the research team were affiliated with the relevant organization, the interviewers did not have supervisory relationships with participants, and measures were taken to minimize the potential influence of these affiliations on participants’ responses.

Finally, findings were aggregated at the national level; therefore, potential differences across provincial ODOs in organizational structures, staffing models, policies, and practices could not be examined. Furthermore, as this study was conducted within the Canadian organ donation system, differences in organizational structures, professional roles, policies, and donation practices may limit the transferability of the findings to other countries and organ donation systems.

## 6. Conclusions

This study identified occupational strain, emotional exhaustion, moral distress, and considerations of leaving the role as important concerns among surveyed Canadian OTDCs. The qualitative findings provided additional context by identifying organizational, workload-related, and ethical circumstances in which these experiences occurred. Together, the findings highlight the importance of considering both individual experiences and organizational contexts when examining OTDC well-being. Future studies should explore organizational factors and evaluate strategies that may support coordinator well-being and baseline knowledge of coordinators.

## Figures and Tables

**Table 1:** Participant Demographics.

Demographic Category	Overall (N = 120)
**Sex**	
Female	98 (82.4%)
Male	21 (17.6%)
*Missing*	1 (0.8%)
**Gender**	
Man	20 (17.2%)
Prefer not to answer	1 (0.9%)
Woman	95 (81.9%)
*Missing*	4 (3.3%)
**Age**	
Mean (SD)	41.8 (9.64)
Median [Min, Max]	40.5 [25.0, 67.0]
*Missing*	4 (3.3%)
**Education (combined)**	
Registered Nurse	117 (97.5%)
Respiratory Therapist	3 (2.5%)
**Years working as OTDC**	
1–6 months	3 (2.5%)
6 months-1 year	16 (13.6%)
More than 1 year	99 (83.9%)
*Missing*	2 (1.7%)
**Years in current OTDC position**	
1–6 months	5 (4.2%)
6 months-1 year	16 (13.6%)
More than 1 year	97 (82.2%)
*Missing*	2 (1.7%)
**Weekly hours as OTDC**	
Mean (SD)	35.7 (12.7)
Median [Min, Max]	37.5 [0, 80.0]
*Missing*	5 (4.2%)
**Difficulty of the last case you managed in terms** **of stress and emotional burden**	
Difficult	45 (37.8%)
Extremely difficult	9 (7.6%)
Fairly difficult	28 (23.5%)
Not difficult at all	37 (31.1%)
*Missing*	1 (0.8%)
**Shift work** **Scheduled day shifts**	
No	16 (13.3%)
Yes	104 (86.7%)
** ** **Scheduled night shifts**	
No	85 (70.8%)
Yes	35 (29.2%)
** ** **On-call night shifts**	
No	12 (10.0%)
Yes	108 (90.0%)
** ** **On-call day shifts**	
No	34 (28.3%)
Yes	86 (71.7%)
**Have you ever seriously considered leaving your current position as an organ and tissue donation coordinator?**	
No	36 (30.0%)
Yes	84 (70.0%)

Note: Sex and gender were collected as separate self-reported demographic variables. Sex referred to participants’ sex assigned at birth, whereas gender referred to participants’ self-identified gender. Percentages were calculated based on available responses for each variable. One respondent reported 0 weekly hours as a coordinator; this value is retained as recorded.

**Table 2:** Maslach Burnout Inventory-Human Services Survey for Medical Personnel (MBI-HSS-MP) measuring burnout in a work–life context.

Burnout Domain	Overall (N = 120)
**Emotional Exhaustion**	
Mean (SD)	28.4 (11.2)
Median [Min, Max]	29.5 [1.00, 49.0]
Missing	2 (1.7%)
**Depersonalization**	
Mean (SD)	7.76 (5.44)
Median [Min, Max]	7.00 [0, 25.0]
Missing	1 (0.8%)
**Personal Accomplishment**	
Mean (SD)	32.1 (6.94)
Median [Min, Max]	32.0 [17.0, 45.0]
Missing	3 (2.5%)

**Table 3:** The Professional Quality of Life Measure (ProQOL).

ProQOL Score	Overall (N = 120)
**Compassion Satisfaction**	
Mean (SD)	36.3 (6.26)
Median [Min, Max]	37.0 [16.0, 49.0]
Missing	7 (5.8%)
**Burnout**	
Mean (SD)	27.6 (6.07)
Median [Min, Max]	27.0 [15.0, 41.0]
Missing	9 (7.5%)
**Secondary Traumatic Stress**	
Mean (SD)	26.4 (7.24)
Median [Min, Max]	26.0 [10.0, 45.0]
Missing	3 (2.5%)

**Table 4:** Independent Samples *t*-Test comparing sex and study variables.

Study Variables	Female (N = 98)	Male (N = 21)	*p*-Value *	Cohen’s d (95% CI)
**MBI-HSS-MP Emotional Exhaustion**				
Mean (SD)	28.3 (11.0)	28.4 (12.7)	0.987 (0.987)	−0.004 (−0.481, 0.473)
Median [Min, Max]	29.5 [1.00, 49.0]	26.0 [2.00, 48.0]		
Missing	2 (2.0%)	0 (0%)		
**ProQOL Compassion Satisfaction**				
Mean (SD)	36.6 (6.51)	34.4 (4.68)	0.104 (0.260)	0.345 (−0.167, 0.857)
Median [Min, Max]	37.0 [16.0, 49.0]	33.5 [25.0, 42.0]		
Missing	4 (4.1%)	3 (14.3%)		
**CD-RISC** **-** **10**				
Mean (SD)	28.4 (5.87)	29.0 (4.26)	0.605 (0.756)	−0.103 (−0.580, 0.375)
Median [Min, Max]	29.0 [10.0, 40.0]	29.0 [23.0, 39.0]		
Missing	2 (2.0%)	0 (0%)		
**ProQOL Secondary Traumatic Stress**				
Mean (SD)	27.3 (6.95)	22.8 (7.63)	0.019 (0.094)	0.639 (0.154, 1.124)
Median [Min, Max]	27.0 [11.0, 45.0]	23.0 [10.0, 43.0]		
Missing	3 (3.1%)	0 (0%)		
**MMD-HP Moral Distress**				
Mean (SD)	111 (80.8)	94.9 (83.3)	0.48 (0.756)	0.202 (−0.347, 0.752)
Median [Min, Max]	88.0 [2.00, 392]	73.0 [11.0, 308]		
Missing	25 (25.5%)	5 (23.8%)		

* Unadjusted *p*-values from Welch’s independent-samples *t*-tests; Benjamini–Hochberg-adjusted *p*-values across the five comparisons in this table are shown in parentheses.

**Table 5:** Independent samples *t*-test comparing years working as coordinator and study variables.

**Study Variables**	**1 Year or Less** **(N = 19)**	**More than 1 Year** **(N = 99)**	***p*** -Value *	**Cohen’s d (95% CI)**
**MBI-HSS-MP Emotional Exhaustion**				
Mean (SD)	24.7 (10.5)	29.0 (11.3)	0.119 (0.199)	−0.377 (−0.877, 0.123)
Median [Min, Max]	23.0 [4.00, 49.0]	31.0 [1.00, 49.0]		
Missing	0 (0%)	2 (2.0%)		
**ProQOL Compassion Satisfaction**				
Mean (SD)	35.1 (6.42)	36.5 (6.23)	0.370 (0.370)	−0.231 (−0.731, 0.269)
Median [Min, Max]	37.0 [16.0, 44.0]	37.0 [22.0, 49.0]		
Missing	0 (0%)	5 (5.1%)		
**CD-RISC** **-** **10**				
Mean (SD)	24.9 (5.47)	29.2 (5.47)	0.005 (0.027)	−0.774 (−1.292, −0.255)
Median [Min, Max]	27.0 [10.0, 31.0]	30.0 [17.0, 40.0]		
Missing	1 (5.3%)	1 (1.0%)		
**ProQOL Secondary Traumatic Stress**				
Mean (SD)	28.4 (7.17)	26.1 (7.25)	0.221 (0.276)	0.306 (−0.194, 0.806)
Median [Min, Max]	29.0 [11.0, 41.0]	26.0 [10.0, 45.0]		
Missing	0 (0%)	3 (3.0%)		
**MMD-HP Moral Distress**				
Mean (SD)	173 (108)	93.5 (66.5)	0.015 (0.037)	1.043 (0.457, 1.629)
Median [Min, Max]	161 [14.0, 392]	82.0 [2.00, 348]		
Missing	4 (21.1%)	26 (26.3%)		

* Unadjusted *p*-values from Welch’s independent-samples *t*-tests; Benjamini–Hochberg-adjusted *p*-values across the five comparisons in this table are shown in parentheses.

**Table 6:** Correlation of the selected scales with age (based on Pearson correlation coefficient).

Study Variables	Age
MBI-HSS-MP Emotional Exhaustion	0.03
ProQOL Compassion Satisfaction	0.10
CD-RISC-10	0.15
ProQOL Secondary Traumatic Stress	−0.11
MMD-HP Moral Distress	−0.15

**Table 7:** Correlation of the selected scales with weekly hours as coordinator (based on the Pearson correlation coefficient).

Study Variables	Weekly Hours
MBI-HSS-MP Emotional Exhaustion	0.25
ProQOL Compassion Satisfaction	0.13
CD-RISC-10	0.08
ProQOL Secondary Traumatic Stress	0.14
MMD-HP Moral Distress	0.00

**Table 8:** Areas of Worklife Survey (AWS) that measures the organizational contexts perceived by employees.

Score	Overall (N = 120)
**Workload**	
Mean (SD)	2.58 (0.690)
Median [Min, Max]	2.60 [1.00, 4.20]
Missing	2 (1.7%)
**Control**	
Mean (SD)	3.30 (0.724)
Median [Min, Max]	3.25 [1.25, 5.00]
Missing	1 (0.8%)
**Reward**	
Mean (SD)	3.18 (0.828)
Median [Min, Max]	3.25 [1.00, 5.00]
Missing	1 (0.8%)
**Community**	
Mean (SD)	3.56 (0.852)
Median [Min, Max]	3.80 [1.00, 5.00]
Missing	0 (0%)
**Fairness**	
Mean (SD)	2.92 (0.808)
Median [Min, Max]	3.00 [1.00, 4.67]
Missing	1 (0.8%)
**Values**	
Mean (SD)	3.48 (0.694)
Median [Min, Max]	3.50 [1.00, 5.00]
Missing	0 (0%)

**Table 9:** Qualitative Illustrations of Workload and Fairness Mismatch.

Theme	Representative Quotes
Workload	“They were just going to leave it overnight because we had our own staffing issues which is a reality of what’s happening across the world right now. […] And because I just chose to stay, because of my own… I just, I wouldn’t have been able to sleep a wink anyways. I would have just been worrying about it. How do you leave your nursing staff, your whatever, but the manager on call was like ‘just leave. I’ll figure it out. I’ll solve it. Someone’ll go in the morning.’ I was like no, no. And then you’re going to go on to me about case length because there’s a whole pile of results that need to be uploaded because they can’t offer out if they don’t have results. So I stayed and worked all night.” (P10)
Fairness	“I gather all the information, I am clear about this, this and this I don’t think are good and this is like not ideal about this donor and then if they decide that they want to go ahead anyway, I’ve done my part to express my concerns. And if they want to proceed then it’s on them, not on me, so.” (P25)

**Table 10:** MMD-HP scale.

MMD-HP Score	Overall (N = 120)
**Moral Distress**	
Mean (SD)	108 (80.8)
Median [Min, Max]	86.0 [2.00, 392]
Missing	30 (25.0%)

**Table 11:** Qualitative Illustrations of Moral Distress Feelings.

Theme	Representative Quotes
Moral Distress	“I go against everything I believe in and again, I feel it more now, because of everything I see around me. Like, healthcare is in crisis.” (P4) “one where sometimes you’re convinced that this is not going to lead into donation, but you still have to do it, is probably the most frequent.” (P5) “And I think moral distress for our team, we have like this whole operating room issues just cause a lot of moral distress because we’ve had surgeons like tell coordinators that they’re purposely not trying to help them. And we’re going to make their recipients die if we don’t do these things. So that causes a lot of moral distress in our team.” (P11) “Here’s an example of moral distress. Staffing issues. Case activity. But before I went away, I was on a case, not in my region, not near my hospital in any way, and my shift was done, and I was leaving on vacation literally the next day. Like out of… I went to [on an international trip]. And so there was nobody coming to relieve me, and things needed to be done. Blood needed to be sent. Results needed to be uploaded. Full-on active case. And I couldn’t leave. Like I just, I morally couldn’t leave my donor and my family, that the work was still there. I knew nobody was coming.” (P10) “Situations where, you know, you’re doing things that don’t seem right. I will say sometimes perhaps now that I’m thinking about it, one of the examples for that for me would have been, you know, sometimes offering families donation when it seems like it’s not going to be something that is going to happen and asking them to go through the process and them being willing to go through the process, kind of having this gut feeling that like I don’t think this is going to go anywhere, those cases don’t feel very good.” (P18)

**Table 12:** Multivariable logistic regression analysis of factors associated with having considered leaving the OTDC position (n = 112).

Characteristic	OR ^1^	95% CI ^1^	*p*-Value
MBI-HSS-MP Emotional Exhaustion	1.14	1.08, 1.21	<0.001
CD-RISC-10	0.96	0.86, 1.06	0.4
ProQOL Secondary Traumatic Stress	0.95	0.88, 1.03	0.3
Years working as OTDC			0.076
1 year or less	—	—	
More than 1 year	3.24	0.88, 12.7	

^1^ OR = odds ratio; CI = confidence interval. ORs are adjusted for all other variables in the model. Complete-case analysis was performed (n = 8 observations excluded from the model owing to missing values in at least one predictor). Model fit statistics: log-likelihood = −51.75; AIC = 113.5; BIC = 127.1; Tjur R^2^ = 0.31; AUC = 0.82; RMSE = 0.38. The AUC of 0.82 indicated good discriminatory ability, and the RMSE of 0.38 indicated relatively good agreement between predicted probabilities and observed outcomes. AIC = Akaike information criterion; AUC = area under the receiver operating characteristic curve; BIC = Bayesian information criterion; RMSE = root mean square error.

**Table 13:** Qualitative Illustrations of Job Turnover Intentions.

Theme	Representative Quotes
Job Turnover	“Oh, absolutely. [Laughs] I, you know, I love the donation job, I feel, like I’ve always known that this is what I’ve wanted to do but it, the hours are draining, they’re exhausting” (P30) “Yes. I have, to the point that I’ve applied for another job and I was going to go back to the ICU actually” (P31) “Yeah. I have spoken to a few people at work about it and I think a lot of us are feeling the same way. Like right now our group has very low, low morale. We’re very yeah, we have a very low morale. I know I’m not the only person thinking about leaving because when I talk to other people they’re thinking about leaving too and applying to other jobs. Yeah.” (P1)

## Data Availability

The datasets presented in this article are not readily available due to ethical and privacy restrictions related to participant confidentiality. Requests regarding the datasets should be directed to the corresponding author.

## Supplementary Materials

The following supporting information can be downloaded at https://www.mdpi.com/article/10.3390/healthcare14213738/s1. Table S1: Tools Description. Table S2: Pearson correlations among continuous scale predictors included in the multivariable logistic regression model. Table S3: Example semi-structured interview guide. Table S4: Comparison of characteristics between participants with missing and non-missing MMD-HP data. Table S5: ProQOL subscale cut-off values (raw score range: 10–50). Table S6: Cut-off values used for interpretation of MBI-HSS-MP subscale scores.

## Abbreviations

The following abbreviations are used in this manuscript:

AIC	Akaike Information Criterion
AUC	Area Under the Receiver Operating Characteristic Curve
AWS	Areas of Worklife Survey
BIC	Bayesian Information Criterion
BO	Burnout
BRiC	Burnout and Resilience in Organ Donor Coordinators
CD-RISC	Connor-Davidson Resilience Scale
CF	Compassion Fatigue
CI	Confidence Interval
COREQ	Consolidated Criteria for Reporting Qualitative Research
CS	Compassion Satisfaction
DP	Depersonalization
EE	Emotional Exhaustion
IT	Information Technology
MBI-HSS-MP	Maslach Burnout Inventory–Human Services Survey for Medical Personnel
MD	Moral Distress
MMD-HP	Measure of Moral Distress for Health Care Professionals
ODOs	Organ Donation Organizations
OR	Odds Ratio
OTDC	Organ and Tissue Donation Coordinator
OTDT	Organ and Tissue Donation and Transplantation
PA	Personal Accomplishment
ProQOL	Professional Quality of Life Measure
RMSE	Root Mean Square Error
RNs	Registered Nurses
SD	Standard Deviation
STS	Secondary Traumatic Stress
STROBE	Strengthening the Reporting of Observational Studies in Epidemiology
