# Supplementary material for: Uncovering Work-Related Issues Among Canadian Organ Donation Coordinators: A National Mixed-Methods Study
Source: Healthcare (Basel). 2026 Nov 3;14(21):3738. doi: 10.3390/healthcare14213738 (PMC13762478; doi:10.3390/healthcare14213738)
Supplement: Supplementary file 1 [file healthcare-14-03738-s001.zip › healthcare-4519818-supplementary.pdf]

Table S1. Tools Description

Evaluation Component	Data Collection Tool	Description of Tool
Burnout	Maslach Burnout Toolkit™ for Medical Personnel [1]	Combines the Areas of Worklife Survey (AWS) and Maslach Burnout Inventory - Human Services Survey for Medical Personnel (MBI-HSS-MP) to measure burnout in a work-life context.
		The MBI-HSS is a well-established list of 22 questions that have been validated for measuring burnout in health professionals, including nurses. The MBI-HSS-MP has also demonstrated good reliability and validity with high internal consistency (Cronbach alpha= 0.80). The three dimensions of burnout are measured in a Likert type scale for emotional exhaustion (EE – nine items), depersonalization (DP – five items), and perceived lack of personal accomplishment (PA – eight items).
		The AWS has also been reported to have high internal consistency (Cronbach alpha= 0.70-0.82), and is used to measure six areas of worklife (workload, control, reward, community, fairness and values) through 28 items in a Likert type scale.
Compassion Fatigue	Professional Quality of Life Measure (ProQOL)[2]	Assesses the negative and positive effects of a career helping others. The ProQOL measures compassion satisfaction (CS) and compassion fatigue (CF), with the latter incorporating two parts to reflect burnout (BO) and secondary traumatic stress (STS). The BO subscale measures feelings of unhappiness, disconnectedness, and insensitivity to the work environment. While the STS subscale relates to the negative feelings caused by work-related fear and trauma, often negative aspects of providing care to those who have experienced extreme or traumatic stressors. This is a reliable scale with high internal consistency (Cronbach alpha for CS= 0.88; BO= 0.75 and STS= 0.81) that has been validated among health professionals.
Moral Distress	Measure of Moral Distress for Health Care Professionals (MMD-HP)[3]	A 27-item scale measuring moral distress levels in health care providers using six parallel versions (adult and pediatric versions for nurses, physicians, and other healthcare professionals). The MMD-HP was developed following a revision to the Moral Distress Scale—Revised (MDS-R), consequently reflecting the most commonly understood reasons for moral distress among those working in acute and critical care settings. The items on the scale are scored using a Likert type scale, providing values for the frequency of occurrence and level of MD related to each question, which

		are then multiplied to receive an overall level of distress. Instead of categorical cut-offs, the instrument measures moral distress on a continuous scale. The total score of MD therefore, ranges from 0 to 432, with increased scores indicating higher levels of MD. The MMD-HP has demonstrated good reliability and validity with high internal consistency (Cronbach alpha= 0.93).
Resilience	Connor-Davidson Resilience Scale (CD-RISC-10) [4]	The 10-item Connor-Davidson Resilience Scale (CD-RISC-10) is a self-report measure of resilience. The scale consists of 10 items, each scored from 0 to 4. Item scores are summed to produce a total score ranging from 0 to 40, with higher scores indicating greater resilience.

The Maslach Burnout Toolkit™ for Medical Personnel combines the Areas of Worklife Survey (AWS) and the Maslach Burnout Inventory–Human Services Survey for Medical Personnel (MBI-HSS-MP) to assess burnout within the context of the work environment. The AWS is a 28-item instrument that evaluates the degree of job-person fit across six areas of worklife: workload, control, reward, community, fairness, and values. Each item is rated on a 5-point Likert scale ranging from 1 (strongly disagree) to 5 (strongly agree), with reverse scoring applied to negatively worded items. For each subscale, scores greater than 3.0 indicate a better match (congruence) between the worker and the workplace, whereas scores below 3.0 indicate a mismatch (incongruence). The MBI-HSS-MP has also demonstrated good reliability and validity with high internal consistency (Cronbach alpha= 0.80). The three dimensions of burnout are measured in a Likert type scale for emotional exhaustion (EE – nine items), depersonalization (DP – five items), and perceived lack of personal accomplishment (PA – eight items). The AWS has also been reported to have high internal consistency (Cronbach alpha= 0.70-0.82), and is used to measure six areas of worklife (workload, control, reward, community, fairness and values) through 28 items in a Likert type scale.

The Professional Quality of Life Measure (ProQOL) assesses the negative and positive effects of a career helping others. The ProQOL measures compassion satisfaction (CS) and compassion fatigue (CF), with the latter incorporating two parts to reflect burnout (BO) and secondary trauma (STS). The BO subscale measures feelings of unhappiness, disconnectedness, and insensitivity to the work environment. While the STS subscale relates to the negative feelings caused by work-related fear and trauma, often negative aspects of providing care to those who have experienced extreme or traumatic stressors. This is a reliable scale with high internal consistency (Cronbach alpha for CS= 0.88; BO= 0.75 and CF= .81) that has been validated among health professionals.

The Measure of Moral Distress for Health Care Professionals (MMD-HP) is a 27-item scale measuring moral distress levels in health care providers using six parallel versions (adult and pediatric versions for nurses, physicians, and other healthcare professionals). The MMD-HP was developed following a revision to the Moral Distress Scale—Revised (MDS-R), consequently reflecting the most commonly understood reasons for moral distress among those working in acute and critical care settings. The items on the scale are scored using a Likert type scale, providing values for the frequency of occurrence and level of MD related to each question, which are then multiplied to receive an overall level of distress. The total score of MD therefore, ranges from 0 to 432, with increased scores indicating higher levels of MD. The MMD-HP has demonstrated good reliability and validity with high internal consistency (Cronbach alpha= 0.93).

Table S2. Pearson correlations among continuous scale predictors included in the multivariable logistic regression model.

Variable	1	2	3
1. MBI-HSS-MP Emotional Exhaustion	1.00	−0.26	0.49
2. CD-RISC score	−0.26	1.00	−0.29

3. ProQOL-STS score	0.49	−0.29	1.00

Table S3. Example semi-structured interview guide

Domain	Example interview questions
Work-related issues	• What do you consider to be the main work-related challenges experienced by organ donation coordinators?<br>• What factors do you believe contribute to these challenges?
Burnout and compassion fatigue	• Do you think burnout and compassion fatigue are problems experienced by you and/or your OTDC colleagues?<br>• If so, could you tell me more about your experiences?<br>• How frequently do you discuss these issues with your colleagues?<br>• What is the nature of these discussions?<br>• In your opinion, what are the main triggers or causes of burnout or compassion fatigue?
Moral distress	• Have you ever completed a task that you felt was contrary to your beliefs or that you felt uncomfortable completing?<br>• Can you think of an example?<br>• Were you ever asked to approach a family at a time you felt was not appropriate?
Work environment and organizational factors	• What workplace or organizational factors contribute to these work-related challenges?<br>• What workplace changes or resources would help coordinators perform their role more effectively?
Job turnover intentions	• Have you ever considered leaving your role as an organ donation coordinator?<br>• What factors influenced that decision?<br>• What changes might encourage coordinators to remain in the profession?

Table S4. Comparison of characteristics between participants with missing and non-missing MMD-HP data.

**Characteristic**	**MMD-HP data missing**, N = 30[1]	**MMD-HP data available**, N = 90[1]	**p-value**[2]
AWS Workload	2.40 (1.80, 3.00)	2.60 (2.20, 3.20)	0.056
Missing	1	1	
AWS Community	3.60 (2.80, 4.20)	3.80 (3.00, 4.00)	0.7
AWS Fairness	3.00 (2.17, 3.50)	2.83 (2.50, 3.50)	0.8
Missing	0	1	
AWS Reward	3.25 (2.50, 3.50)	3.25 (2.75, 3.75)	0.3
Missing	0	1	
AWS Values	3.75 (3.25, 4.00)	3.50 (3.00, 4.00)	0.5
AWS Control	3.25 (3.00, 4.00)	3.50 (2.75, 3.75)	0.8
Missing	0	1	
MBI-HSS-MP Emotional Exhaustion	31 (25, 37)	29 (18, 36)	0.3
Missing	1	1	
MBI-HSS-MP Depersonalization	8.0 (3.0, 12.0)	7.0 (3.0, 12.0)	>0.9
Missing	1	0	
MBI-HSS-MP Personal Accomplishment	33 (27, 37)	32 (27, 38)	0.7
Missing	0	3	
Use of any substance to manage job stress, 2019 (pre-pandemic)			0.7
Daily or frequently	3 (10.0%)	13 (14.4%)	
Never	8 (26.7%)	28 (31.1%)	
Occasionally	19 (63.3%)	49 (54.4%)	
Use of any substance to manage job stress, 2020			0.8
Daily or frequently	11 (36.7%)	37 (41.1%)	
Never	8 (26.7%)	26 (28.9%)	
Occasionally	11 (36.7%)	27 (30.0%)	
Use of any substance to manage job stress, 2021			0.6
Daily or frequently	8 (26.7%)	32 (35.6%)	
Never	8 (26.7%)	25 (27.8%)	
Occasionally	14 (46.7%)	33 (36.7%)	

Ever used alcohol	29 (96.7%)	84 (93.3%)	0.7
Ever used tobacco products	10 (34.5%)	37 (42.5%)	0.4
Missing	1	3	
Ever used marijuana	18 (60.0%)	38 (43.7%)	0.12
Missing	0	3	
Ever used prescription drugs	0 (0.0%)	6 (7.0%)	0.3
Missing	1	4	
Ever used illicit drugs	5 (16.7%)	6 (7.0%)	0.15
Missing	0	4	
ProQOL Compassion Satisfaction	37 (33, 41)	36 (33, 40)	0.7
Missing	3	4	
ProQOL Burnout	26.5 (23.0, 30.0)	27.0 (23.0, 33.0)	0.6
Missing	4	5	
ProQOL Secondary Traumatic Stress	29 (24, 31)	26 (21, 32)	0.2
Missing	1	2	
CD-RISC-10	31.0 (24.0, 32.0)	28.5 (25.0, 31.5)	0.7
Missing	0	2	
PSS score	20.0 (15.0, 25.0)	19.0 (13.0, 23.0)	0.3
Missing	3	3	
Sex			0.9
Female	25 (83.3%)	73 (82.0%)	
Male	5 (16.7%)	16 (18.0%)	
Missing	0	1	
Gender			>0.9
Man	5 (16.7%)	15 (17.4%)	
Prefer not to answer	0 (0.0%)	1 (1.2%)	
Woman	25 (83.3%)	70 (81.4%)	
Missing	0	4	
Age (years)	40 (35, 47)	41 (35, 49)	0.6
Missing	2	2	
Educational background			0.7
Other (specify)	0 (0.0%)	1 (1.1%)	
Registered Nurse	30 (100.0%)	86 (95.6%)	
Respiratory Therapist	0 (0.0%)	3 (3.3%)	

Years working as OTDC			0.8
1-6 months	1 (3.3%)	2 (2.3%)	
6 months-1 year	3 (10.0%)	13 (14.8%)	
More than 1 year	26 (86.7%)	73 (83.0%)	
Missing	0	2	
Years in current OTDC position			>0.9
1-6 months	1 (3.3%)	4 (4.5%)	
6 months-1 year	3 (10.0%)	13 (14.8%)	
More than 1 year	26 (86.7%)	71 (80.7%)	
Missing	0	2	
Weekly hours as OTDC	40 (30, 40)	37 (30, 40)	0.4
Missing	0	5	
Difficulty of last case managed in terms of stress and emotional burden			0.7
Difficult	12 (40.0%)	33 (37.1%)	
Extremely difficult	3 (10.0%)	6 (6.7%)	
Fairly difficult	8 (26.7%)	20 (22.5%)	
Not difficult at all	7 (23.3%)	30 (33.7%)	
Missing	0	1	
Ever seriously considered leaving current OTDC position	23 (76.7%)	61 (67.8%)	0.4
Years working as OTDC (combined)			0.8
1 year or less	4 (13.3%)	15 (17.0%)	
More than 1 year	26 (86.7%)	73 (83.0%)	
Missing	0	2	
Years in current OTDC position (combined)			0.5
1 year or less	4 (13.3%)	17 (19.3%)	
More than 1 year	26 (86.7%)	71 (80.7%)	
Missing	0	2	
Difficulty of last case managed (combined)			0.6
Difficult or Extremely difficult	15 (50.0%)	39 (43.8%)	
Fairly difficult	8 (26.7%)	20 (22.5%)	

Not difficult at all	7 (23.3%)	30 (33.7%)	
Missing	0	1	

[1]Median (Q1, Q3) for continuous variables; n (%) for categorical variables. Percentages were calculated from non-missing responses; missing values are reported as counts. AWS = Areas of Worklife Survey; CD-RISC-10 = 10-item Connor-Davidson Resilience Scale; MBI-HSS-MP = Maslach Burnout Inventory–Human Services Survey for Medical Personnel; MMD-HP = Measure of Moral Distress for Health Care Professionals; OTDC = organ and tissue donation coordinator; ProQOL = Professional Quality of Life Measure.

[2]Wilcoxon rank-sum test for continuous variables; Fisher's exact test or Pearson's chi-squared test, as appropriate, for categorical variables.

Table S5. ProQOL subscale cut-off values (raw score range: 10–50).

**ProQOL Subscale (Raw Score Range: 10–50)**	**Low**	**Moderate (Average)**	**High**
**Compassion Satisfaction (CS)**	≤ 22	23 – 41	≥ 42
**Burnout (BO)**	≤ 22	**23 – 41**	≥ 42
**Secondary Traumatic Stress (STS)**	≤ 22	23 – 41	≥ 42

Table S6. Cut-off values used for interpretation of MBI-HSS-MP subscale scores.

Subscale Dimension	Low	Moderate	High
**Emotional Exhaustion (EE)**	0 – 16	17 – 26	≥ 27
**Depersonalization (DP)**	0 – 6	7 – 12	≥ 13

**Personal Accomplishment (PA)***	≥ 39	32 – 38	0 – 31

*Personal accomplishment is scored in the opposite direction to emotional exhaustion and depersonalization: lower scores indicate greater burnout-related impairment. Cut-off values are those reported in the MBI Manual (3rd ed.) [1].

References

1. Maslach, C.; Jackson, S.E.; Leiter, M.P. *Maslach Burnout Inventory Manual*, 3rd ed.; Consulting Psychologists Press: Palo Alto, CA, USA, 1996.
2. Stamm, B.H. *The Concise ProQOL Manual*, 2nd ed.; ProQOL.org: Pocatello, ID, USA, 2010.
3. Epstein, E.G.; Whitehead, P.B.; Prompahakul, C.; Thacker, L.R.; Hamric, A.B. Enhancing Understanding of Moral Distress: The Measure of Moral Distress for Health Care Professionals. *AJOB Empir Bioeth.* **2019**, *10*, 113–124
4. Connor, K.M.; Davidson, J.R.T. Development of a new resilience scale: The Connor-Davidson Resilience Scale (CD-RISC). *Depress Anxiety.* **2023**, *18*, 76–82.
